# A case vignette study to refine the target group of an intermediate care model: the Acute Geriatric Community Hospital

**DOI:** 10.1007/s41999-024-00947-6

**Published:** 2024-02-28

**Authors:** Eline D. Kroeze, Aafke J. de Groot, Susanne M. Smorenburg, Janet L. Mac Neil Vroomen, Anneke J. A. H. van Vught, Bianca M. Buurman

**Affiliations:** 1 Section of Geriatric Medicine, Department of Internal Medicine, Amsterdam Public Health Research Institute, Amsterdam UMC, University of Amsterdam, Amsterdam, The Netherlands; 2 Department of Medicine for Older People, Amsterdam Public Health Research Institute, Amsterdam UMC, Vrije Universiteit Amsterdam, Amsterdam, The Netherlands; 3 HAN University of Applied Sciences, School of Health Studies, Research Group Organisation of Healthcare and Services, Nijmegen, The Netherlands; 4 Canisius Wilhelmina Hospital, Nijmegen, The Netherlands

**Keywords:** Case vignettes, Qualitative research, Intermediate care, Acute geriatric unit, Admission criteria, Older people

## Abstract

**Purpose:**

To refine the admission criteria of the Acute Geriatric Community Hospital (AGCH) by defining its target group boundaries with (geriatric) hospital care and other bed-based intermediate care models in the Netherlands.

**Methods:**

A qualitative study consisting of a three-phase refinement procedure with case vignettes. Physicians, medical specialists, nurse practitioners, and physician assistants in hospitals (*n* = 10) or intermediate care facilities (*n* = 10) in the Netherlands participated. They collected case vignettes from clinical practice (phase one). The referral considerations and decisions for each case were then documented through surveys (phase two) and two focus groups (phase 3). For thematic data analysis, inductive and deductive approaches were used.

**Results:**

The combination of medical specialist care (MSC) and medical generalist care (MGC), is unique for the AGCH compared to other intermediate care models in the Netherlands. Compared to (geriatric) hospital care, the AGCH offers a more limited scope of MSC. Based on these findings, 13 refined admission criteria were developed such as ‘The required diagnostic tests to monitor the effectiveness of treatment are available at the AGCH’. Besides admission criteria, additional clinical and organizational considerations played a role in referral decision-making; 10 themes were identified.

**Conclusion:**

This case vignette study defined the target group boundaries between the AGCH and other care models, allowing us to refine the AGCH admission criteria. Our findings may help to determine the required competencies of the interdisciplinary AGCH team and to develop triage instruments. The identified consideration themes can be used as conceptual framework in further research. The findings may also be of interests for healthcare systems outside the Netherlands who aspire to design integrated care for older people closer to home.

## Key summary points

**Aim:**

Refining the admission criteria of the Acute Geriatric Community Hospital (AGCH) by defining its target group boundaries with (geriatric) hospital care and other bed-based intermediate care models.

**Findings:**

The integration of medical specialist care (MSC) and medical generalist care (MGC) is unique for the AGCH compared to other intermediate care models and (geriatric) hospital care in the Netherlands. Based on these findings, 13 refined admission criteria were developed. Also, 10 additional consideration themes were identified.

**Message:**

Because care is shifting from hospitals to the community, target group boundaries between (intermediate) care models should be continuously (re)defined; based on our experiences, we recommend using case vignettes as this yields rich and practical input.

## Introduction

Older patients who are acutely hospitalized are at risk of adverse health outcomes such as disability, morbidity, mortality, and readmission [1–6]. To avoid these negative outcomes and to help older adults live longer independently at home, several countries have implemented intermediate care models [7–10]. Intermediate care involves a broad range of temporary services that integrate care, ensure continuity and quality of care, and promote recovery at the interface between hospital and home, care home, primary care, and community services. These services are provided close to home, support early discharge, and reduce readmission to acute care [11, 12]. Examples of intermediate models are acute geriatric units [10, 13, 14], subacute care units in nursing homes [9, 15], community hospitals [16, 17], intensive outpatient follow-up [18] and hospital at home [19]. These models focus on preventing functional decline and frailty in older adults.

Acute geriatric units (AGUs) provide acute medical care services for older adults with Ambulatory Care Sensitive Conditions [20], such as heart failure, pneumonia, COPD, and frailty. When chronic conditions worsen or ‘minor’ acute events occur, patients receive acute care and early rehabilitation at the AGU instead of in the hospital [21]. AGUs appear similar to community hospitals in Northern Europe [22, 23] and the United Kingdom [16, 17] where mixed specialist and generalist expertise is provided. Nonetheless, patients typically stay longer at community hospitals (11–58 days [16, 17, 22, 23]) than at AGUs (10–14 days [21]) because community hospitals also provide post-acute care. Therefore, community hospitals and AGUs are distinct models of care.

The AGU model pioneered in Spain in 2012, and is a potential alternative to conventional hospitalization for older adults [10, 13]. The AGU model was recently implemented in Amsterdam (referred to as the Acute Geriatric Community Hospital (AGCH)) and has proven effective [14]. A prospective controlled observational study showed that patients admitted to the AGCH had a lower risk of readmission or death within 90 days after discharge than patients admitted to the hospital [24]. Patient satisfaction was high [25] and incident rates for delirium were lower [26] compared with control groups. These findings have motivated multiple stakeholders to scale up the model to other sites in the Netherlands.

To scale up the AGCH model, the criteria that determine admission to the AGCH over other intermediate care models and (geriatric) hospital care needed to be refined to ensure the ‘right’ patients are admitted [27]. Therefore, the aim of this study was to refine the AGCH admission criteria by defining how its targeted patient group differs from (geriatric) hospital care and other bed-based intermediate care models in the Netherlands.

## Methods

### The Dutch context

Because of rising expenses in long-term care (LTC), the Dutch government reformed the healthcare system in 2015 with a shift to the non-residential setting. This involved stricter criteria for LTC admission, with nursing homes only admitting patients needing continuous care [28]. The government also introduced short-term residential care (STRC) for patients needing short-term care that does not require hospital admission, but who cannot be treated at home [7]. STRC was, next to geriatric rehabilitation (GR), the second bed-based intermediate care model available in the Netherlands. A third model, the AGCH, was recently introduced as an alternative to hospitalization for older patients with frailty. Care at the AGCH is provided by an interdisciplinary team of nurses (with experience in hospital and community care) and paramedics (see Table 1). At the AGCH in Amsterdam, care is coordinated by a nurse practitioner, physician assistant or medical resident in collaboration with a hospital geriatrician. Other sites are exploring whether the elderly care physician (ECP) can fulfill this role. ECPs are primary care experts in geriatric medicine and traditionally provide medical generalist care services in nursing homes. Geriatricians (or other medical specialists) traditionally provide medical specialist care services in the hospital (see Supplement 1.3). The feasibility of a nurse-led AGCH is also being explored, where care would be coordinated by the nurse practitioner or physician assistant.

**Table 1:** Bed-based intermediate care models in the Netherlands

**Bed-based intermediate care for frail older adults in the Netherlands**		
**Geriatric revalidation **	**Short-term residential stay **	**Acute Geriatric Community Hospital **
*Definition:* Post-acute multidisciplinary (para)medic care for older and frail patients, including those with pre-existing functional decline or specific care needs [45, 46]	*Definition:* Medical care for older adults with general health problems that do not require specialist care nor geriatric rehabilitation, but whose treatment and care needs cannot be met at home [7]	*Definition:* (Sub)acute specialized geriatric medical care for older patients with frailty [14]
*Goal: * To optimize functional capacities and support societal participation despite impairments, so that frail and/or older individuals can return home and live independently in the community [32]	*Goal:* Recovery so that older adults can return home and live independently in the community [7]	*Goal:* To provide medical specialist care for frail older adults in an adapted environment, so they can return home, live independently in the community and hospital (re)admissions are prevented [14]
*Admission route:* Admission from hospital, ED, or home [32] Referral by a medical specialist or ECP using a comprehensive geriatric assessment [32]	*Admission route:* Admission from home, ED, or hospital [7] Referral by GP or medical specialist [7]	*Admission route:* Admission from ED [14] Referral by a medical pecialist [14]
*Admission criteria* [47]: (i) Medical stability (ii) Multidisciplinary rehabilitation needs (iii) Frailty and/or multimorbidity (iv) Motivation/preference to undergo rehabilitation treatment (v) A cognitive and physical status that allows participation in geriatric rehabilitation Targeted diagnoses: (1) stroke, (2) elective orthopedics, (3) trauma surgery (e.g., hip fractures), (4) amputations, and (5) other disorders (neurodegenerative diseases, oncological diseases, COPD, cardiac failure, internal- and multi-system failure)	*Admission criteria *[7]: No guidelines or targeted patient groups	*Admission criteria (before 2023)* [14]: (i) Older patient with an acute medical problem that requires hospitalization, such as pneumonia or exacerbation of chronic conditions such as heart failure (ii) Geriatric conditions (e.g. delirium, cognitive/functional impairment, falls) (iii) Hemodynamic stability (iv) No complex diagnostic testing needed such as CT or MRI scans during admission (v) Return to previous living situation expected in 14 days

See Supplement 1 for the complete table (with staffing, coordinating practitioners, treatment components and funding)

*AGCH *Acute Geriatric Community Hospital, *COPD* chronic obstructive pulmonary disorder, *CT* computed tomography, *ED* emergency department, *MRI* magnetic resonance imaging, *STRC* short-term residential care

### Study design

**Fig. 1:**
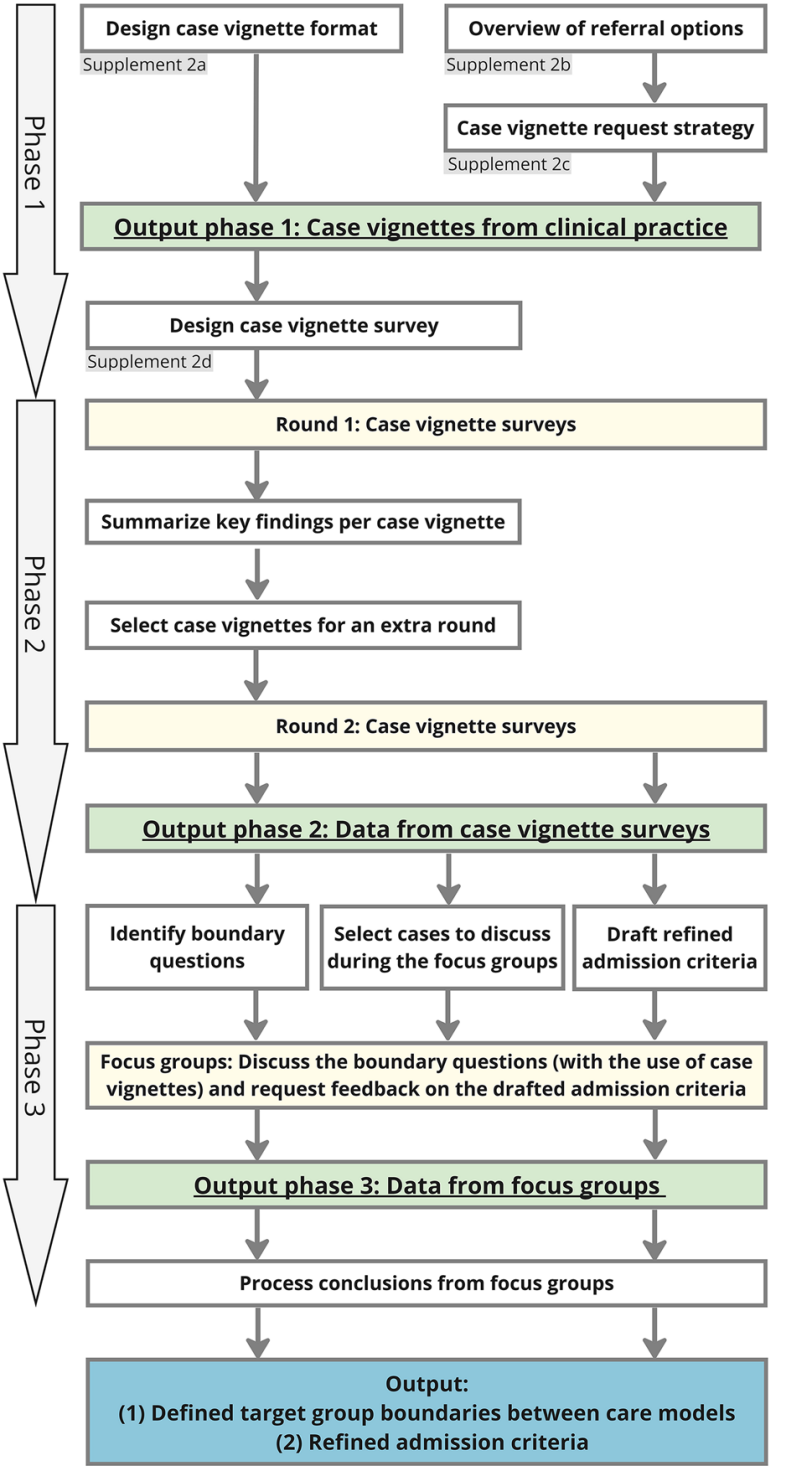
The three-phase refinement procedure with case vignettes

To our knowledge, no guidelines and methods exist to refine admission criteria or to distinguish the target groups for different care models. Therefore, we developed and followed a target group refinement procedure of three phases, in which vignettes played a central role (Fig. 1). Case vignettes were chosen as this is valid, inexpensive, practical, and well-suited method for assessing the decision-making processes of healthcare professionals [29, 30]. We used the COREQ-checklist [31] to ensure all items relevant to reporting qualitative research were included (see Supplement 4). The study protocol was submitted to the Amsterdam University Medical Centre’s Medical Ethics Research Committee (file number 2023.0477). The need for official approval was waived as the Medical Research Involving Human Subjects Act did not apply. Written consent was obtained from all participants.

### Participants and research team

The participants (*n* = 20) were ECPs, medical specialists, nurse practitioners, and physician assistants involved in the implementation of an AGCH in their region and/or who were familiar with referral decision-making to intermediate and (geriatric) hospital care. A purposive sampling method was used to obtain participants from various professional backgrounds and regions. Participants were recruited via an e-mail explaining the goal of the study. The research team consisted of a PhD student with a health economic background (EK, MSc), a PhD student who worked as an ECP and specialized in GR (AG, MD), the AGCH program manager (SS, PhD), an associate professor with expertise on ‘aging in place’ policies (JM, PhD), an associate professor with expertise on ‘the right care at the right place’ (AV, PhD), and a professor in acute geriatric care (BB, PhD).

### Data collection and analysis

#### Phase 1: Co-creating case vignettes

During phase one, participants collected case vignettes from clinical practice for a specific care model (AGCH, STRC, GR, (geriatric) hospital care) or for a ‘gray area’ between the AGCH and other care models. First, a format was designed for the case vignettes (Supplement 2a) (EK, SS) based on the somatic, psychological, functional, and social geriatric domains. Also an overview of bed-based referral options for older adults after admission to the emergency department was created (EK, SS, BB). With this overview (Supplement 2b) and with the stakeholders’ questions in mind on the boundaries between the AGCH and other care models, a case vignette request strategy (Supplement 2c) was drafted (EK, SS). Participants were asked to collect 1–3 cases from clinical practice and to submit these cases (anonymized) using Microsoft Word. The data were e-mailed to the researcher (EK) and converted into a JPG file. An example of a case vignette is provided in Supplement 2d.

#### Phase 2: Case vignette surveys

In phase 2, a survey was conducted to gather information on the referral decisions and considerations of at least three participants per case vignette in three contexts: 1) the current regional context in which the participant practices, 2) the current regional context plus the AGCH as available care model, and 3) the regional context in which all bed-based intermediate and (geriatric) hospital care options are continuously available. The rationale behind this was to elicit information about the current referral practice in the region, the possible shift in patient flows after implementation of an AGCH, and the referral decision considered appropriate by participants in an ideal regional situation. A concept version of the survey was discussed during an one-hour online feedback session (with participants E, K, and G in Microsoft Teams) and refined accordingly (EK, SS). The final survey (SurveyMonkey) included 12 questions per case vignette (seven open and five closed questions; see Supplement 2d). In the first survey round, each participant received an unique link with three case vignettes to review (3 × 12 questions).

Data were analyzed after the survey deadline. The survey data from round one were first merged into one data document, and case vignettes with diverse referral outcomes and/or with fewer than three referral decisions were selected (EK, SM) for round two. Some participants indicated that information was missing so they could not make a fully informed decision. This missing information was retrieved via e-mail from the case provider and added to the case vignette so that participants had this information for the second survey round and the focus groups.

After round two, EK and SS summarized the results per case vignette and read all qualitative data. Then they analyzed the data manually and separately 1) to identify the boundary questions (i.e., questions participants raised on the boundaries between the AGCH and other care models) and 2) to identify desirable changes to the AGCH admission criteria. After discussing this analysis, SS and EK created an initial coding scheme for the data analysis in Atlast.ti (see 2.3.4) and drafted the first version of the refined admission criteria.

#### Phase 3: Focus groups

In phase three, the boundary questions were discussed during two focus groups using the case vignettes. The three goals of these sessions were 1) to answer the boundary questions, 2) to explore healthcare professionals’ considerations during referral decision-making, and 3) to member-check the refined admission criteria. Data were considered saturated when the boundary questions from focus group participants and researchers were answered. Ultimately, two one-hour focus groups were conducted online in Microsoft Teams. Both were led by SS and observed by EK, and were audio-recorded and transcribed verbatim. The AGCH program leaders were also invited to participate. The conclusions from the focus groups were summarized and sent to the participants for comments and/or corrections.

#### Data analysis

After phase three, all qualitative data from the case vignette surveys and focus groups were analyzed in Atlas.ti (software version 9.0.24.0) using a deductive and inductive approach. The a priori categories were drawn from the initial coding scheme formulated by EK and SS after phase two. As our objective was to also identify the considerations made by healthcare professionals during referral decision-making, three extra a priori categories were added: clinical considerations (3A), organizational considerations (3B), and weighing consideration themes (3C). Data on theme 3A were analyzed deductively based on de Groot et al.’s framework [32] of clinical triage factors in acutely hospitalized older patients. Relevant new categories were included. Data on themes 3B and 3C were coded inductively. After all data were coded, EK and AG discussed the coding scheme. If the data were not sufficient to support the a priori categories, they were removed. EK and AG agreed on the final codes and categories and the overarching themes (see Supplement 3, Table S5).

## Results

### Participants and case vignettes

Twenty healthcare professionals from nine (pioneer) regions participated (see Table 2): 12 provided patient cases (phase one), 17 were survey respondents (phase two), and six participated in one or two focus groups (phase three). Of the 28 case vignettes requested during phase one, 23 were delivered (see table S3, Supplement 2c). These 23 cases were reviewed by the participants of survey round one, and six of these 23 cases were reviewed in round two by 10 participants who had not reviewed the case vignette before. The survey results (phase two) are illustrated per case vignette in Table S4 (Supplement 3).

**Table 2:** Participant characteristics and participation

Participant characteristics				Participation during …		
Code	Profession	Setting	Region	Phase 1 Case collection	Phase 2 Case vignette survey (round 1 + 2)	Phase 3 Focus groups
A	ECP	Intermediate care facility	1	✓	✓ ✓	
B	CG	Hospital	1	✓	✓	
C	ECP	Intermediate care facility	2	✓		
D	ECP	Intermediate care facility	3		✓ ✓	
E	MS	Hospital	3	✓	✓ ✓	
F	ECP	Hospital	3		✓	
G	CG	Hospital	4	✓	✓ ✓	✓ ✓
H	ECP	Intermediate care facility	5	✓	✓ ✓	✓ ✓
I	CG	Hospital	5	✓	✓ ✓	✓ ✓
J	ECP	Intermediate care facility	6	✓	✓ ✓	
K	MS	Hospital	7	✓	✓	✓
L	CG	Intermediate care facility	7		✓ ✓	✓
M	NP	Hospital	7		✓	
N	CG	Intermediate care facility	8	✓		
O	MS	Hospital	8			✓
P	MS	Hospital	9		✓	
Q	NP	Skilled nursing facility	9	✓	✓ ✓	
R	PA	Skilled nursing facility	9		✓	
S	PA	Skilled nursing facility	9		✓	
T	NP	Hospital	9	✓	✓ ✓	
				*N* = 12	*N* = 17 | *N* = 10	*N* = 6 | *N* = 3

*ECP* elderly care physician, *CG* clinical geriatrician, *MS* medical specialist (other than CG), *NP* nurse practitioner, *PA* physician assistant

### Discussing the boundaries between the AGCH and other care models

The boundary questions raised by survey participants (phase 2) were categorized into 10 themes. There were questions on A) the completeness of diagnostics and treatment plan before transfer to the AGCH, B) the maximum monitoring and treatment possible at the AGCH, C) the handling of treatment risks at the AGCH, D) medical specialist(s) (in consult) at the AGCH, E) functional decline and the rehabilitation goals mandatory for AGCH admission, F) geriatric syndromes mandatory for AGCH admission, G) referral decision-making for patients with delirium, H) the maximum treatment complexity at other intermediate care models, I) the maximum observation and treatment intensity at other intermediate care models, and J) the observation of cognitive functioning at other intermediate care models. During focus group 1 (phase 3), the boundary between the AGCH and (geriatric) hospital care was discussed, and boundary questions A–G were addressed using case vignettes 5, 9, 11 and 16. During focus group two, the boundary between the AGCH and other bed-based intermediate care models was discussed, and boundary questions G–J were addressed using case vignettes 4, 10, 15 and 17.

#### The AGCH versus (geriatric) hospital care

An indication for medical specialist care (MSC) is needed for admission to the AGCH and a (geriatric) hospital ward. The AGCH offers a limited scope for MSC, which was defined by the participants as “*low-complex specialized geriatric care from the hospital perspective*”*.* Unlike the (geriatric) hospital ward, the AGCH provides these ‘low-complex’ MSC services with medical generalist care (MGC) services (such as early rehabilitation).

Based on the case vignette discussions, treatments that are minimally available at the AGCH are intravenous medication, oxygen, nebulizing, tube feeding, and airway suctioning. Participants considered (geriatric) hospital care more appropriate for older patients who were hemodynamically unstable and needed intense monitoring (such as continuous vital signs monitoring) or complex diagnostics to finalize or monitor the treatment plan. Finally, patients should not be admitted if there are substantial (treatment) risks for which care could not be provided at the AGCH, such as an intensive care (IC) risk and no IC treatment limitation decision.

Diagnostics is more limited at the AGCH than at a general hospital, so participants argued that the diagnoses and treatment plan must be clear before AGCH admission. Nevertheless, the AGCH can monitor the effectiveness of treatment and can adjust the treatment plan if needed. Diagnostics available at the AGCH include point-of-care testing, electrocardiograms, bladder scans, pulse oximetry, and simple x-ray. Additional diagnostics might be available depending on the AGCH location. If an AGCH is situated inside or in close proximity to the hospital, the staff may be able to use the diagnostic resources of the hospital (e.g., CT/MRI scan or quick laboratory testing). Whether patients with infectious diseases can be admitted to the ACGH depends on the quarantine possibilities. Lastly, the AGCH needs a closed ward to accommodate patients at risk of wandering. These patients do not receive appropriate care at hospital, so the focus groups advocated for AGCH locations to admit patients with MSC indications and risk of wandering.

#### AGCH versus bed-based intermediate care

Focus group participants concluded that the boundary between the AGCH and other bed-based intermediate care models is defined by an indication for MSC. Unlike other bed-based intermediate care models in the Netherlands, the AGCH uniquely combines MSC and MGC elements as the medical specialist is involved in the care provided at the AGCH. In other Dutch bed-based intermediate care models, the ECP or GP is responsible and no medical specialist is involved, so only patients requiring MGC are admitted.

Our case vignette survey and focus group results revealed that several specialized services are provided at some STRC/GR facilities. These services include intravenous treatment or a peripherally inserted central catheter line. Focus group participants argued that this is desirable as long as 1) the ECP or GP and the nursing team are capable and have the expertise to deliver these (acute) specialized services and/or interventions without the involvement of a medical specialist, 2) the high intensity of treatment and monitoring can be met by the ECP or GP and the nursing team, and 3) the care is properly reimbursed. According to the participants, GR (unlike the AGCH) prioritizes the rehabilitation needs of patients. Therefore, rehabilitation goals are set and the treatment needed to reach these goals starts at GR admission.

### The refined AGCH admission criteria

After phase 2, the refined AGCH admission criteria were drafted according to the survey data analysis. These criteria were then further refined based on feedback from the focus group. Ultimately, 13 refined AGCH admission criteria were defined (see Table 3).

**Table 3:** Initial and refined AGCH admission criteria

Initial AGCH admission criteria [14]	Refined admission criteria (2023)
(i). Older patient with an acute medical problem that requires hospitalization, such as pneumonia or exacerbation of chronic conditions such as heart failure (ii). Hemodynamic stability (iii). No need for complex diagnostic testing such as CT or MRI scans during admission (iv). Return to previous living situation expected in 14 days (v). Geriatric conditions (e.g. delirium, cognitive/functional impairment, falls)	1. The patient has an **acute medical care need** (e.g., exacerbation of chronic conditions such as heart failure, COPD, diabetes mellitus; acute ‘minor events’ like respiratory tract infections such as pneumonia, urinary tract infections; observations and pain relief after fall without fracture). 2. The patient has **one or more geriatric syndromes** (e.g., functional impairments/decline; cognitive impairments such as delirium and dementia; fall risk; malnutrition). 3. The **medical specialist must be involved** in the treatment. 4. The patient is **hemodynamically stable**. 5. The **diagnoses and treatment plan are clear** before AGCH admission. 6. The required **treatment (complexity)** is possible at the AGCH. Treatment options: intravenous medication, oxygen, nebulizing, tube feeding, airway suctioning.* 7. The **diagnostic tests** needed to monitor the effectiveness of treatment are available at the AGCH. Diagnostic options: electrocardiogram, POC testing (e.g. hemoglobin, blood gas, calcium, CRP, troponin, INR, glucose, ACT, blood ketone, creatine), bladder scan, pulse oximeter, x-ray.* 8. The required **continuous nursing assistance** and **monitoring intensity** is feasible at the AGCH.* 9. **Risks (of treatment)** lead to consequences which are treatable at, or in close proximity to, the AGCH (or the patient has a treatment limitation policy such as no intensive care).* 10. In case of the treatment of patients with **wandering** symptoms, **behavioral problems,** and **infectious diseases** (e.g., influenza, COVID**), safe admission** to the AGCH is possible.* 11. Return to previous living situation (or admission to residential LTC) is expected **within 14 days**. 12. **Early rehabilitation and activation** to prevent functional decline will benefit the patient 13. A **low stimuli environment** will benefit the patient (e.g., delirium preventive)

*ACT* activated clotting time, *AGCH* Acute Geriatric Community Hospital, *COPD* chronic obstructive pulmonary disease, *CRP* C-reactive protein, *CT* computed tomography, *INR* international normalized ratio, *MRI* magnetic resonance imaging, *LTC* long-term care, *POC* point-of-care

*Further specification per AGCH location is needed: 6) additional treatment options, 7) additional diagnostic options, 8) feasible monitoring intensity (at night), 9) consequences which are not treatable at the AGCH (or in close proximity to the AGCH), 10) patients for whom safe admission to the AGCH is not possible

The participants concluded that the combination of MSC and MGC is unique in the Netherlands to the AGCH model of intermediate care. Therefore, a criterion (3) was added that states that the medical specialist must be involved in the treatment to meet the acute medical care needs. How much involvement is required will depend on the competency of the ECP or GP and/or the nursing team. Furthermore, criterion 3 was refined into a more specific criterion (5): *‘the diagnoses and treatment plan are clear’.* In addition, criteria 6–10 were formulated so they can be further specified by (future) AGCH facilities. The (extra) treatment possibilities (criteria 6, 9, and 10) and diagnostic tests available (criterion 7) as well as the feasible intensity of monitoring (criterion 8) should be defined by each AGCH. The AGCH intervention was designed to minimize the risk of functional decline, delirium, and readmission. Therefore, criterion five was refined into ‘*the patient has one or more geriatric syndromes*’ (criterion 2). In addition, criteria 12 and 13 were added: *‘early rehabilitation and activation to prevent functional decline and a low stimuli environment are of benefit for the patient’*.

### Considerations during referral decision-making

The AGCH admission criteria were not the only decisive element during referral decision-making. Other considerations can be divided into clinical and organizational considerations (Table 4). Seven clinical consideration categories were identified: demographic, somatic, cognitive and mental status, social, mobility and functional status, multi-domain, and patient preferences. Focus group participants concluded that referral decision-maker formulates a decision by weighing these clinical considerations, in agreement with other professionals (e.g., a medical specialist, ECP, GP, nurse practitioner, physician assistant).

**Table 4:** Consideration themes and (sub)categories

	Consideration categories	Consideration subcategories
Clinical considerations*	Demographic	Age Characteristics housing
	Somatic**	Clinical status Diagnosis-related Plan for treatment
	Cognitive and mental status	Dementia Delirium (or risk of delirium) Hallucinations (Repellent) behavior Other cognitive impairment(s) Receptive to instruction(s) for rehabilitation Awareness of illness Momentary well-being
	Social***	Caregiver situation Social system of patient Involvement of case manager (dementia)
	Mobility and functional status	Mobility Functional independence (ADL/iADL) Functional decline (ΔADL/ΔiADL) Barthel index Premorbid activity limitation High fall risks Patient’s capacity for training
	Multi-domain****	Case complexity Judgment concerning probability of returning to own home LTC indication (and on waiting list) Patient has a rehabilitation need Self-management ability (Reduced) self-care
	Patient preferences***	Limited treatment policy Wishes of the patient with respect to LTC admission Follow-up care possible at same facility? (fewer transfers)
Organizational considerations*	Micro-level: Care process at the (intermediate) care location	Diagnostic procedures Observational procedures Execution of treatment (plan) Capability and expertise of the intermediate care team Multidisciplinary care Coordination of care Environmental factors
	Meso-level: Regional organization of (acute) care for older people	Availability of intermediate care beds (and which models) Possibilities of acute care in an outpatient or home setting Availability of home care Availability of support material/instruments to use at home Time of day when referral decision-making takes place
	Macro-level: Healthcare system factors	Patient’s insurance Pressure to discharge early Reimbursement for provided care (not sufficient) Costs for society/efficiency of care Admission to care model possible directly after ED visit?

*Renamed, in the de Groot et al. [32]: clinical and organizational triage factors ** renamed, in de Groot et al. [32]: diagnoses, syndromes; *** new category; ****renamed, in de Groot et al. [32]: multi-domain tools and measures)

*ADL* activities of daily living, *iADL* instrumental activities of daily living, *LTC* long-term care, *ED* emergency department

Organizational considerations influencing the referral decision were categorized into micro-, meso- and macro-level factors. Micro-level factors concerned the care process at the (intermediate) care locations (e.g., the capabilities and expertise of the intermediate care team, and whether the diagnostic and (multidisciplinary) treatment needs can be provided safely). Meso-level considerations concerned the manner in which acute care for older people is organized in the region (e.g., the availability of beds and the possibilities of providing acute geriatric care in an outpatient/home setting). Macro-level factors concerned the organization of the national healthcare system (e.g., whether the care is sufficiently reimbursed). When these organizational factors are barriers for admission, healthcare professionals indicated that they would choose the ‘*next best option*’.

## Discussion

Our findings have uncovered differences between target groups of the AGCH, (geriatric) hospital care, and other bed-based intermediate care models in the Netherlands. Based on these findings, 13 refined admission criteria have been formulated. We found that referral decision-making is not only a matter of ‘ticking off’ the admission criteria but also involves multiple clinical and organizational considerations. Because care is shifting from hospitals to the community, target group boundaries between (intermediate) care models should be continually (re)defined, particularly when a new model is implemented. Drawing from our experiences, we recommend using a three-phase procedure with case vignettes as this yields rich and practical input. The comprehensive description of intermediate care models and their target group boundaries can also be of interest for healthcare systems outside the Netherlands who aspire to design integrated care for older people closer to home.

Based on our findings, we anticipate that implementing the AGCH will induce several changes for healthcare professionals and organizations. First, the AGCH has to be implemented in synergy with other interventions along the continuum of integrated care for older people closer to home. When combined specialist and generalist medical services are not required for the acute geriatric patient, admission to another intermediate care model, such as geriatric rehabilitation or short-term residential care is more appropriate. These should preferably be available without barriers, so that waiting days are minimal. Second, we expect that hospitals will have a more limited role in providing MSC care to geriatric patients as low-complex care (from the hospital perspective) will be moved to the AGCH. To facilitate this shift, the referring healthcare professionals should trust that patients can be safely treated at the AGCH. Having AGCH ambassadors at the emergency department could promote referral [27]. Third, intermediate care facilities will have new responsibilities. Based on the 13 refined (and per AGCH location specified) admission criteria, the competencies that need to be present in the interdisciplinary team to provide AGCH care should be discussed. Training and education will also be needed to give the nursing team the necessary knowledge and skills to provide acute geriatric MSC [27]. Fourth, the allocation of (coordinating) tasks and responsibilities for AGCH care should be discussed by the ECP and hospital geriatrician. A shared vision by these specialists for older people on their roles in acute care would facilitate this. Preferably, the need for intensive collaboration between these specialists and other healthcare professionals (such as the GP) along the whole continuum of care for frail older persons (from prevention to post-acute care) is highlighted in this vision. To stimulate intensive collaboration, joint training programs and/or the implementation of (intermediate) care models like the AGCH where specialist and generalist expertise is mixed, may function as catalysts. When nurse practitioners and physician assistants at the AGCH provide the care of the ECP (and/or hospital geriatrician), the legal consequences of transferring responsibilities should be clear [33]. Fifth, nursing home organizations will need to adjust their operations, logistics, and billing to provide acute care 24/7 because patient turnover is higher at the AGCH (x̄ length of stay 10 days [26]) than at the STRC (x̄ 34 days [34]) or GR (x̄ 43 days [35]). Finally, those providing specialized services at intermediate care facilities should use the 13 refined admission criteria to determine which of these services can be defined as AGCH care. The expected length of stay (max. 14 days) will play a crucial role in this process.

In accordance with previous research, we found that referral decision-making involves more than just ‘ticking off’ the admission criteria. Eligibility is assessed by considering seven patient factor themes: demographics, somatic status, cognitive and mental status, social situation, mobility and functional status, multi-domain criteria, and patient preference. These characteristics arise from a comprehensive (geriatric) assessment, so correspond to the criteria of multi-domain tools to identify rehabilitation needs or predict rehabilitation outcomes [36, 37]. When the preferred referral decision is not possible because of micro-, meso-, or macro-level organizational factors, healthcare professionals deviate to the second-best option. Similar to the findings of Pearson et al. [11], we found that regional health system characteristics (meso) could bound care options for service users. In agreement with Buntin et al. [38], we also found that healthcare professionals deviate to the second-best option when the facility characteristics (micro) are not appropriate. Previous research has also reported on the limitations that national healthcare regulations and insurance policies (macro) put on referral options [39–42]. This corresponds with our finding that healthcare professionals consider whether reimbursement for the delivered care would be sufficient if the patient were admitted to the clinically optimal referral option. Noteworthy is that ‘the wish of family caregivers’, ‘telemedicine’ and/or ‘IT systems’ were not identified as themes, whilst we can imagine that this could have an influence on the decision-making process in practice.

The target group was refined in several cycles of action and reflection, and four main lessons were learned. First, case vignette surveys allowed us to collect superficial data, but did not provide an in-depth understanding of the boundaries between care models. Therefore, we added participant interaction through focus groups, which sharpened and redefined the individual responses [43]. Second, case vignettes were a useful tool for surveys and focus groups because they ‘speak the language of healthcare professionals’ and enable concrete discussion about boundaries. Third, case vignettes situated in the ‘gray areas’ between care models provided the most valuable input for answering the boundary questions and refining the admission criteria. Therefore, the target group refinement procedure should focus on collecting and reviewing these case vignettes. Fourth, fellow researchers with medical knowledge and practical referral experience are vital for chairing the focus groups or accurately analyzing the data.

Our research objective was to refine the AGCH admission criteria by defining the target group boundaries between (geriatric) hospital care and other bed-based intermediate care models in the Netherlands. Therefore, we did not collect case vignettes for other intermediate care model boundaries (i.e., STRC versus GR). We also collected only one case vignette of a patient with palliative and end of life care needs, whilst this is often a reality of (post-)acute care for older people. This constraints our ability to develop an intermediate care triage tool for Dutch referral decision-makers. To create such a comprehensive triage tool, additional case vignettes for the target group boundaries between STRC and GR should be discussed. Another limitation is scope of questions we addressed during the case vignette questionnaire and focus groups. This limitation could have attributed to not identifying ‘the wish of family caregivers’, ‘telemedicine’ and/or ‘IT systems’ as themes from our qualitative data. We also wanted a diverse participant group for the focus groups (phase three). As the second focus group was arranged at short notice (three weeks before it started): only three participants from two different regions attended. Therefore, information bias may have influenced the results. To correct for information bias, the conclusions from the focus groups were member checked. We also tested for coding accuracy by carrying out spot checks (AG) on the analyzed data. Table 5 illustrates all strategies that were applied to establish trustworthiness and rigor of our findings; these were categorized according to Lincoln and Guba’s four-dimension criteria framework [44]. All participants were actively involved in an AGCH pioneer site. Since SS, AV and EK are coordinators of the the pioneer sites program, they already had relationships with most participants. We believe that these prior relationships positively encouraged participants to share their experiences. Moreover, all participants worked for organizations at the forefront of moving care from the hospital to the community. Therefore, we believe we captured those developments that influence target group boundaries between care models. This study also reformulated ten categories that healthcare professionals consider beyond the formal admission criteria when deciding where to admit older adults for intermediate care. We renamed two and added two clinical consideration categories to de Groot et al.’s framework [32] and formulated three organizational categories (micro-, meso-, and macro-level factors) (see Table 4). These 10 categories for consideration (or ‘triage factors’ as defined in Groot et al.’s framework) can be used as a conceptual framework for further research. Finally, we have ensured relevance and transferability of our findings to systems outside the Netherlands by providing a rich descriptions of the case vignette method, the intermediate care models, and the national context in which they are implemented (see supplementary file 1).

**Table 5:** Key four-dimension criteria strategies adapted from Lincoln and Guba [44]

Rigor criteria	Purpose	Strategies applied
Credibility	To establish confidence that the results (from the perspective of the participants) are true, credible, and believable	We ensured that the researchers had the required knowledge and research skills (e.g., medical knowledge) to perform their roles Member check of the AGCH admission criteria by focus group participants
Dependability	To ensure the findings of this qualitative inquiry are repeatable if the inquiry occurred within the same cohort of participants, coders, and context.	We developed a track record of the data collection process Rich description of the study and the target refinement procedure (with the use of a flow diagram) We tested for coding accuracy by spot checks (AG)
Confirmability	To extend the confidence that the results would be confirmed or corroborated by other researchers.	We applied several triangulation techniques (methodological, data source, investigators, and theoretical) EK and SM implemented reflexive meetings after phase 2 and 3 EK and AG implemented reflexive meetings after the additional data analysis
Transferability	To extend the degree to which the results can be generalized or transferred to other contexts or settings.	Purposeful sampling of study participants We defined data saturation when the boundary questions were clarified for the participants and researchers

## Conclusion

This case vignette study defined the boundaries between target groups of the AGCH, (geriatric) hospital care, and other bed-based intermediate care models in the Netherlands and refined the AGCH admission criteria. These criteria can help determine necessary competencies for the interdisciplinary AGCH team. The findings also provide more guidance for appropriate triage and can support the development of a triage instrument. However, additional research on the boundary between STRC and GR is needed to develop a comprehensive ‘intermediate care triage tool’ for (Dutch) referral decision-makers. This tool should also consider that referral decision-making is not just a matter of ‘checking off’ admission criteria but (also) involves clinical and organizational considerations. The consideration themes identified can be used as conceptual framework in further research. The findings of this study may as well be of interest for healthcare systems outside the Netherlands who aspire to design integrated care for older people closer to home.

## Supplementary Information

Below is the link to the electronic supplementary material.

Supplementary file1 (PDF 231 KB)

Supplementary file2 (PDF 362 KB)

Supplementary file3 (PDF 244 KB)

Supplementary file4 (PDF 3055 KB)

## Data Availability

The data generated and analyzed during this study are not publicly available but are available from the corresponding author on reasonable request.
