# Supplementary material for: A case vignette study to refine the target group of an intermediate care model: the Acute Geriatric Community Hospital
Source: Eur Geriatr Med. 2024 Feb 28;15(4):977–89. doi: 10.1007/s41999-024-00947-6 (PMC11377459; doi:10.1007/s41999-024-00947-6)
Supplement: Supplementary file 2 — Supplementary file2 (PDF 362 KB) [file 41999_2024_947_MOESM2_ESM.pdf]

Supplement 2 Instruments

Supplement 2a Case vignette format

Table S2. Case vignette format

**Name (fictional)**	**…**
Age	**…**
Submitted to the ED by	**…**
Submitted to the ED with the following problems(s)	**…**
Assessment at the ED on the 4 geriatric domains	
- Medical (comorbidity, polyfarmacy, nutritional condition)	**…**
- Psychological (cognition, mood, delirium)	**…**
- Functional (ADL, iADL, mobility, falling)	**…**
- Social (support system, coping)	**…**
Wishes with respect to treatment (advance care planning)	**…**
Diagnoses at the ED	**…**
Treatment plan	**…**
Expected intensity monitoring and treatment	**…** hours per week
Risks with respect to treatment	**…**

Additional questions asked to case provider per case vignette

1. Did the patient have an indication for hospital care?
2. What was the outcome of referral decision-making for this patient?
3. Which clinical triage factors were taken into account during the referral decision-making process? (e.g. intensity of monitoring and treatment, experience of healthcare professionals at intended location, medical risks etc.).
4. Which organisational triage factors were taken into account during the referral decision-making process? (e.g. availability STRC beds, time of discharge, financial considerations etc.).
5. It the funding for the care provided at the care type to which the patient was submitted (answer Q2), sufficient?
6. If only clinical triage factors played a role during the referral decision-making process; would the outcome of your triage assessment been different?

Supplement 2b Overview of referral options for older adults after ED admission in the Netherlands

Based on exploratory conversations and meetings with:

- Programme managers of the AGCH project in the pioneer sites
- Healthcare professionals involved in the implementation of an AGCH in their region
- Stakeholders on the national level: the ministry of Health Welfare and Sport (VWS), the Health Care Authority (NZa), the National Health Care Institute (ZIN), Health Insurers the Netherlands (ZN) and the branch organisation for organisations who are active in care and support for elder people (Actiz).

… and an analysis of the following documents during desk research:

- Amsterdam UMC (2022) In- and exclusion criteria AGCH & flow-chart ED to admission [in Dutch] [intern document]
- Zorgcirkel and Dijklander (2022) In- and exclusion criteria of the AGCH in Purmerend [in Dutch] [intern document]
- Evean (2022) Patient categories and financing labels Recura Revalidation [in Dutch] [intern document]
- Significant (2016) Referral decision tool for STRC admission [in Dutch] [link]
- Verenso (2013) Triage instrument Geriatric Revalidation [in Dutch] [link]
- Health Insurance Act Article 2.5c Geriatric Revalidation (2016, 6 October) Overheid.nl Visited 1 march 2022 [link]
- NZA (2017) Policy rules for STRC – BR/REG – 18118 [in Dutch] [link]
- NZA (2023) Current innovation experiments. Visitied 1 march 2022 [in Dutch] [link]
- ZonMW (2022) Evaluation of pioneer sites and experiments GR, STRC and MCSP [in Dutch] [link]
- ZZC Midden Nederland (2022) Process for care coordination short term admissions MGC [link]

.

... the AGCH model of care was presented in context with other models of care for older adults after ED admission (see figure 2). We hypothesized that two 'grey areas' exist: one between the AGCH and short term models of care for older adults within the MGC domain and one within the MSC domain; between the AGCH and hospital admission.

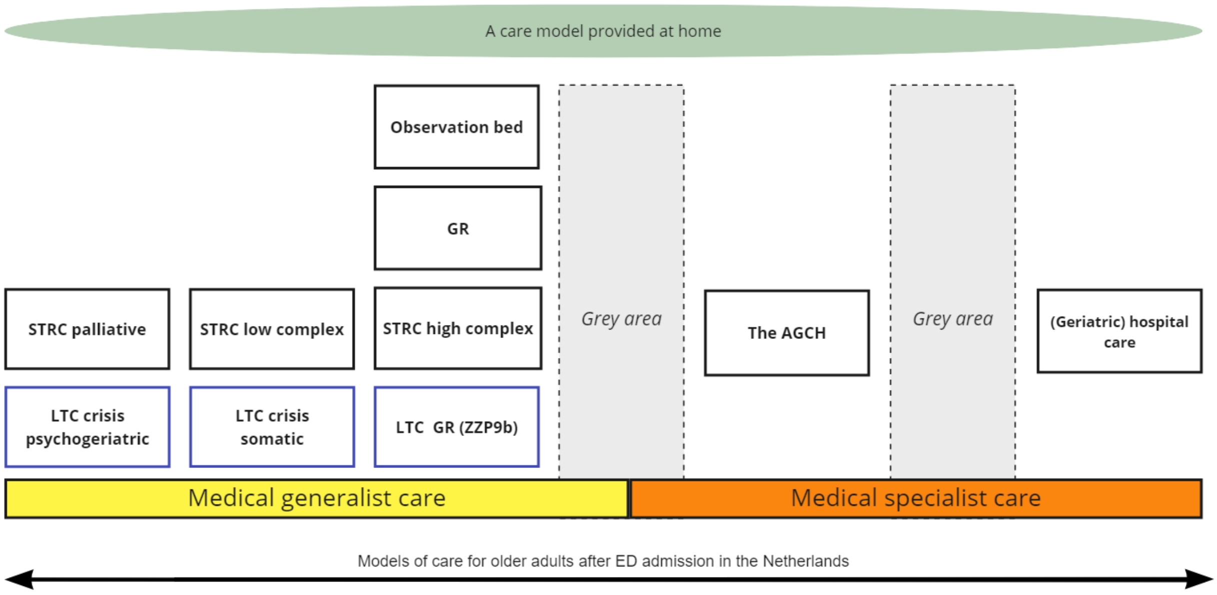

Fig. S1. Models of care for older adults after ED admission in the Netherlands (2022)

Supplement 2c Case vignette request strategy

Table S3. Case request strategy

Region	Case requests	Professional	Delivered	Case name
1	1. A patient with a long-term care indication who was admitted to the delirium department of the intermediate care facility for elderly in the region.<br>2. A patient who was admitted to the hospital, but who would have been a suitable patient for the AGCH if there were one in the region.	A + B	1. ✓<br>2. ✓	17. Lime<br>18. Orange
2	1. A patient who was admitted to the hospital, but who would have been a suitable patient for the AGCH if there were one in the region.<br>2. An older medically stable patient who was in need of intravenous AB treatment<br>3. An older patient who received the Hospital of Home model of care	C	1. X<br>2. X<br>3. ✓	22. Lychee
3	1. A patient who was admitted to the hospital, but who would have been a suitable patient for the AGCH if there were one in the region<br>2. An older medically stable patient who needed oxygen therapy<br>3. A patient who was admitted to an experiment observation bed in the region	E (	1. ✓<br>2. ✓<br>3. ✓	6. Pomegranate<br>7. Kaki<br>8. Cherry
4	1. A patient who was admitted to the hospital, but who would have been a suitable patient for the AGCH if there were one in the region<br>2. A patient who was admitted to an STRC low complex in the region<br>3. A patient who was admitted to the geriatrics department of the hospital and would also be admitted there, if there were an AGCH in the region	G	1. ✓<br>2. ✓<br>3. ✓	19. Papaja<br>20. Mango<br>21. Doerian
5	1. A patient with (a risk for) delirium who was admitted to the AGCH<br>2. An older medically stable patient who needed intravenous fluid administration<br>3. A patient who was admitted to the geriatrics department of the hospital and would also be admitted there, if there were an AGCH in the region	H + I	1. ✓<br>2. ✓<br>3. ✓	9. Pear<br>10. Medlar<br>11. Nectarine
6	1. A patient who was admitted to the hospital, but who would have been a suitable patient for the AGCH if there were one in the region<br>2. A patient who was admitted to the geriatrics department of the hospital and would also be admitted there, if there were an AGCH in the region<br>3. An older patient who received the Hospital of Home model of care	J	1. X<br>2. X<br>3. ✓	23. Guave
7	1. A patient for whom it was debatable whether he/she had a medical specialist care (/hospital) indication or medical generalist care (/STRC HC) indication.<br>2. A patient who was admitted to the hospital, but who would have been a suitable patient for the AGCH if there were one in the region	K	1. ✓<br>2. ✓	15. Lemon<br>16. Mandarin
8	1. A patient who was admitted to an experiment observation bed in the region<br>2. A patient who was admitted to the hospital, but who would have been a suitable patient for the AGCH if there were one in the region.<br>3. A patient which was first admitted to STRC HC but whose 'label' was switched to GR once the patient could strain his/her muscles/body	N	1. ✓<br>2. ✓<br>3. ✓	12. Peach<br>13. Chestnut<br>14. Walnut
9	1. A typical AGCH patient who was admitted at the AGCH in the region<br>2. A patient in need of a high intensity of monitoring and medical treatment at the AGCH in the region<br>3. A patient with delirium who was admitted to the AGCH in the region<br>4. A patient who was admitted to the AGCH, but should have been admitted to STRC if there was sufficient bed capacity in the region<br>5. A patient with atypical clinical presentation(s) and high fall risk	Q + T	1. ✓<br>2. ✓<br>3. ✓<br>4. ✓<br>5. ✓	1. Apple<br>2. Plum<br>3. Mulberry<br>4. Fig<br>5. Almond

Supplement 2d Referral decision questionnaire

<insert JPG case vignette>

Fig. S2. Example case vignette

**Name**	**Mister Pear tree**
**Age**	75
**Submitted to the ED by**	General Practitioner
**Submitted to the ED with the following problems(s)**	Urinary tract infection, decompensatio cordis, trauma capitis
**Assessment at the ED on 4 geriatric domains**	
**- Medical**	eGFR 25ml/min, Parkinson's disease, DM type II, had several urinary tract infections in the past, atrial fibrillation
**- Psychological**	Impulsive person and limited awareness of his own health deficits<br>Susceptible to delirium (experienced in the past)
**- Functional**	ADL: Help with washing, bathing and dressing.<br>iADL: Informal caregivers helps with laundry and groceries. No home care<br>Mobility: Very poorly due to deterioration Parkinson's disease and urinary tract infection
**- Social**	Widow: his wife died 1 year ago. Committed family; his nephew, niece and sister-in-law are the informal caregivers.
**Wishes with respect to treatment**	Restrictions to treatment. No reanimation, no intensive care.<br>Admission to hospital: yes
**Diagnoses at the ED**	Urinary tract infection, atrial fibrillation, constipation, general weakness: patient is not able to mobilize himself, hallucinations, altered levels of consciousness.
**Treatment plan**	Intravenous antibiotics<br>Start oral anticoagulantia for atrial fibrillation<br>Administration of laxative<br>Low stimuli (delirium preventive) environment<br>Movement sensor in case of wandering<br>Physiotherapy<br>Ergo therapy
**Expected intensity monitoring and treatment**	>3h per week
**Risks with respect to treatment**	Sepsis, progressive deterioration Parkinson, high fall risk

1. Does the patient have an indication for medical specialist care?
   - ☐ Yes
   - ☐ No
   - ☐ That is not clear (yet), because: …………

2. In the current regional context, to which care model would you refer the patient?
   - ☐ Inpatient geriatrics department
   - ☐ Other department in the hospital (e.g. internal medicine, cardiology, pulmonary medicine)
   - ☐ Hospital at home
   - ☐ The AGCH (in Amsterdam/Purmerend)
   - ☐ Geriatric revalidation
   - ☐ STRC high complex
   - ☐ STRC low complex
   - ☐ STRC palliative
   - ☐ LTC crisis psychogeriatric
   - ☐ LTC crisis somatic
   - ☐ LTC ZZP9b (GR)
   - ☐ Other choice, that is: …..

3. Which healthcare professionals should be involved with this patient at this care model?
   a) Which healthcare professional should take care of the patient? : …..
   b) Under whose supervision?: …..
   c) Which healthcare professionals should be asked in consult?: …..

4. If the AGCH was one of the care model options in the region, would you refer the patient to the AGCH?
   - ☐ Yes
   - ☐ No
   - ☐ The AGCH is already an option in my region (Amsterdam/Purmerend) …………

5. Which healthcare professional should be involved with this patient at the AGCH?
   [*answer with '-' to 5a-c if you answered 'no' to question 4*]
   [*answer 'idem see answers to question 2' to 5a-c if you answered 'the AGCH' to question 2*]
   a) Which healthcare professional should take care of the patient? : …..
   b) Under whose supervision?: …..
   c) Which healthcare professionals should be asked in consult?: …..

6. Why is/isn't the patient a suitable patient for the AGCH?: …..

7. Which medically related (/clinical) considerations did you take into account during the referral decision-making process (i.e. Q2)?
   a) Staff competency and treatment options at the facility: …..
   b) Feasibility to meet the required intensity of monitoring and treatment: …
   c) (Clinical) risks: …..

8. Which considerations with regards to the patient personal preferences, social system and living conditions did you take into account during the referral decision-making process in the current regional context (i.e. Q2)?
   [*e.g. the patient's (functional) independence, social network, caregivers, living situation*]
   …..

9. Could you explain how organisational triage factors influence the result of your referral decision-making process in the current regional context (i.e. Q2)?
   [*e.g. available options of care models in the region, time at which referral decision-making takes place, capacity and availability of beds, expertise at location etc.]*
   …..

10. Do financial considerations have a role in the referral decision-making process in the current regional context (i.e. Q2)?
   [*e.g. (requirements for) financial compensation, insurance of patient or other financial incentives*.]
   …..

11. Would you choose a different care model for the patient in a hypothetical context where all care models are 24/7 available, always have capacity and when organisational triage factors and financial considerations don't influence referral decision-making?
   [*i.e. when only medical (q7) and personal (care) organizational considerations (q8) play a role*]
   - ☐ Inpatient geriatrics department
   - ☐ Other department in the hospital (e.g. internal medicine, cardiology, pulmonary medicine)
   - ☐ Hospital at home
   - ☐ The AGCH
   - ☐ Geriatric revalidation
   - ☐ STRC high complex
   - ☐ STRC low complex
   - ☐ STRC palliative
   - ☐ LTC crisis psychogeriatric
   - ☐ LTC crisis somatic
   - ☐ LTC ZZP9b (GR)
   - ☐ Other choice, that is: …..

12. The case vignettes contains sufficient (clear) information to make a referral decision:
   - ☐ Yes
   - ☐ No, the following is not clear: ….

13. Email address and name (for verification purposes): ….
