# Supplementary material for: A case vignette study to refine the target group of an intermediate care model: the Acute Geriatric Community Hospital
Source: Eur Geriatr Med. 2024 Feb 28;15(4):977–89. doi: 10.1007/s41999-024-00947-6 (PMC11377459; doi:10.1007/s41999-024-00947-6)
Supplement: Supplementary file 3 — Supplementary file3 (PDF 244 KB) [file 41999_2024_947_MOESM3_ESM.pdf]

Supplement 3 Results

Table S4. Collected case vignettes and key results (after phase 2 (EK, SM)).

Case description		Key results after case vignette questionnaires (round 1 + 2)		
**Case name**	**Key words**	**Referral decision (context 3 optimal regional situation)**	**Identified boundary questions (categories)**	**Discussed during working session**
1. Apple	Dyspnoea, influenza A, delirium, IV antibiotics, 24/7 monitoring, need additional diagnostics	AGCH: 3x<br>STRC HC: 1x		
2. Plum	Dyspnoea, influenza A, suspicion dec cordis after packed cells	AGCH: 3x<br>STRC HC: 1x<br>HOSP: 1x	(B) Monitoring and treatment intensity AGCH<br>(I) Monitoring and treatment intensity other intermediate care models	
3. Mulberry	Delirium, dementia, urinary tract infection (UTI), IV antibiotics	AGCH: 3x<br>STRC HC: 1x (LTC financing label)		
4. Fig	Decreased mobility, bed confinement. No clear diagnosis.	AGCH: 1x<br>STRC HC: 1x<br>STRC LC: 2x	(H) Treatment complexity at other intermediate care models<br>(I) Monitoring and treatment intensity other intermediate care models<br>(J) Observation cognitive functioning	Session 2 AGCH vs. intermediate care
5. Almond	Suspicion delirium, psychotic features, delusions. UTI, urinary retention hyponatraemia	AGCH: 2x<br>HOSP: 1x	(A) Completeness diagnostics and treatment plan<br>(B) Monitoring and treatment intensity AGCH<br>(D) Medical specialist(s) in consult at the AGCH	Session 1 AGCH vs. hospital
6. Pomegranate	Resp. insufficiency type 1 (COVID), IV antibiotics, high need for O2 (NIV), high mortality risk	AGCH: 1x<br>HOSP: 2x		
7. Kaki	Suspicion pneumonia (infl. A), O2 need (3 liter), elevated CK	AGCH: 4x		
8. Cherry	Urosepsis, IV antibiotics, additional diagnostics/lab, renal impairment, low intensity monitoring, treatment restrictions	AGCH: 4x		
9. Pear	General weakness, UTI, atrial fibrillation, IV antibiotics, oral anticoagulant therapy, various specialists in consult	AGCH: 6x<br>HOSP: 1x	(A) Completeness diagnostics and treatment plan<br>(B) Monitoring and treatment intensity AGCH<br>(C) Handling of treatment risks at the AGCH<br>(D) Medical specialist(s) in consult at the AGCH<br>(E) Functional decline and rehabilitation goals	Session 1 AGCH vs. hospital
10. Medlar	Somatic cause fall: UTI & dehydration. Vertebral compress-ion (L2). IV antibiotics, IV fluids.	AGCH: 2x<br>STRC HC: 2x	(H) Treatment complexity at other intermediate care models<br>(I) Monitoring and treatment intensity other intermediate care models<br>(J) Observation cognitive functioning	Session 2 AGCH vs. intermediate care
11. Nectarine	General weakness, dyspnoea, dec. cordis, hyponatraemia, hypokalaemia, elevated CRP, fluid retention IV furosemide, need additional diagnostics	AGCH: 3x<br>HOSP: 1x	(A) Completeness diagnostics and treatment plan<br>(B) Monitoring and treatment intensity AGCH<br>(C) Handling of treatment risks at the AGCH<br>(D) Medical specialist(s) in consult at the AGCH<br>(E) Functional decline and rehabilitation goals	Session 1 AGCH vs. hospital

12. Peach	Subacute pain left inguinal area; femur bone cyst. multidisciplinary care demand, physical therapy, need additional diagnostics (MRI)	AGCH: 1x<br>STRC HC: 2x (O/W 1x observation)<br>Home: 2x	(H) Treatment complexity at other intermediate care models<br>(I) Monitoring and treatment intensity other intermediate care models<br>(J) Observation cognitive functioning	
13. Chestnut	General weakness, mild hypoxemia, COVID, infl. A, IV fluids and O2 need	AGCH: 1x<br>STRC HC: 1x<br>HOSP: 2x	(F) Geriatric syndromes	
14. Walnut	Hip fracture (THR right) after fall. Not ready for physiotherapy as adhesive has to dry.	GR: 3x (O/W 1x first period not trainable and STRC HC label)		
15. Lemon	Mixed hyperactive, apathetic delirium, no somatic substrate, wandering, need additional diagnostics	AGCH: 1x<br>ELV HC: 2x	(G) Delirium<br>(I) Monitoring and treatment intensity other intermediate care models<br>(J) Observation cognitive functioning	Session 2 AGCH vs. intermediate care
16. Mandarin	Delirium, delusions, deep hyponatraemia, complex specialist care need, various specialists in consult	AGCH: 1x<br>HOSP: 3x	(A) Completeness diagnostics and treatment plan<br>(B) Monitoring and treatment intensity AGCH<br>(D) Medical specialist(s) in consult at the AGCH	Session 1 AGCH vs. hospital
17. Lime	Decreased mobility, UTI, COVID, dyspnoea, delirium, wandering, vascular dementia (LTC ZZP5 indication), need additional observation/ diagnostics	AGCH: 2x<br>STRC HC: 2x (O/W 2x LTC financing label)	(G) Delirium<br>(H) Treatment complexity at other intermediate care models<br>(I) Monitoring and treatment intensity other intermediate care models<br>(J) Observation cognitive functioning	Session 2 AGCH vs. intermediate care
18. Orange	Dyspnoea, bacterial pneumonia, exacerbation COPD, hyponatraemia, IV antibiotics glucose and saline, O2 need, monitoring vital function 3x per day.	AGCH: 3x		
19. Papaya	Contusion left ribs due to fall, influenza A, mild hyponatraemia, suspicion UTI, O2 need, IV diuretics, monitoring electrolyte imbalances,	AGCH: 3x<br>Hospital: 1x	(A) Completeness diagnostics and treatment plan<br>(D) Medical specialist(s) in consult at the AGCH<br>(E) Functional decline and rehabilitation goals	
20. Mango	General weakness after COVID, phatic disorders after ICVA. Need for further observation/ diagnostics to assess whether return home is feasible.	STRC LC: 2x<br>STRC HC:1x	(H) Treatment complexity at other intermediate care models<br>(J) Observation cognitive functioning	
21. Durian	Progressive dyspnoea, severe emphysematous destruction lung tissue (COPD), dec. cordis. O2 need (NIV), IV furosemide, IV AB, risk hypercapnia, need for more diagnostics/lab (neurofibromatosis, electrolytes, blood gas, CT thorax)	HOSP: 3x		
22. Lychee	Dyspnoea, progressive cardiac failure, COPD, IV diuretics, monitoring kidney function, no treatment restrictions.	AGCH: 3x<br>HOSP: 2x (O/W 1x hospital at home)		
23. Guave	Dyspnoea, low saturation, pneumonia due to COVID. O2 need, monitoring vital signs, no treatment restrictions.	HOSP: 3x (O/W 1x hospital at home)		

Table S5. Coding table

Themes	Categories boundary questions / considerations	Subcategories boundary questions / considerations
1. AGCH admission criteria	Current AGCH admission criteria	Older patient with an acute medical problem that requires hospitalization<br>Adequate hemodynamic stability<br>No need for complex diagnostic tests<br>Return to previous living situation expected within 14 days<br>Geriatric syndromes
	Additions to the AGCH admission criteria	Admission via ED<br>Clarity and agreement on the diagnosis and treatment<br>24/7 medical specialist care and monitoring is indicated<br>The medical treatment is low to medium complex<br>The (medical) monitoring intensity is low<br>Risks (of treatment); lead to consequences which are treatable at the AGCH location<br>Early rehabilitation and activation is beneficial for the patient<br>A low stimuli environment is of benefit for the patient<br>Admission of patients with specific symptoms and diseases
2A.Triage ambiguities<br>AGCH vs. bed-based geriatric hospital care	(A) Completeness of diagnostics and treatment before transfer to the AGCH	Diagnostics at AGCH<br>Case complexity at AGCH<br>Treatment plan (not) definitive before admission AGCH<br>Lab at AGCH<br>Medical oxygen at AGCH
	(B) Maximum monitoring and treatment possibility at the AGCH	Monitoring vital signs at AGCH<br>Maximum monitoring intensity at AGCH<br>Maximum treatment intensity at AGCH
	(C) Handling of treatment risks at the AGCH	Treatment risks during admission at AGCH<br>No limited treatment policy (and admission AGCH)
	(D) Medical specialist(s) in consult at the AGCH	Whether medical specialist should be in consult<br>Maximum number of medical specialists in consult at AGCH
	(E) Functional decline and rehabilitation goals mandatory for AGCH admission	Maximum score (ADL) functioning for AGCH admission?<br>Hospital associated functional decline expected<br>Rehabilitation goal(s)<br>Prognosis concerning return home is unclear<br>LTC indication<br>Palliative care needs
	(F) Geriatric syndromes mandatory for AGCH admission	Age<br>Frailty<br>Geriatric syndromes
2B. Triage ambiguities<br>AGCH vs. bed-based intermediate care (STRC, GR)	(G) Referral decision-making for patients with delirium	Delirium: when hospital vs. intermediate care indication?<br>Referral decision-making when delirium + hospital indication<br>Referral decision-making when delirium + hospital indication + no AGCH (with locked ward) available<br>Referral decision-making when delirium + no hospital indication<br>Referral decision-making when delirium + no hospital indication + LTC indication
	(H) Maximum treatment complexity at other intermediate care models,	Specialized medical care (AGCH) versus general medical care (STRC and GR)<br>Treatment expertise at STRC and GR<br>Nursing procedures at STRC and GR

Kroeze ED, de Groot AJ, Smorenburg SM, Macneil Vroomen JL, van Vught AJ, Buurman BM (2024) A case vignette study to refine the target group of an intermediate care model: the Acute Geriatric Community Hospital. European Geriatric Medicine

	(I) Maximum observation and treatment intensity at other intermediate care models	Monitoring and treatment intensity at STRC and GR<br>Treatment hours possible at STRC, GR and AGCH<br>Possibilities with regards to lab at STRC and GR
	(J) Observation of cognitive functioning at other intermediate care models	Not clear at hospital/ED whether return home is possible or LTC indication should be issued<br>Diagnostics at STRC observation bed<br>Monitoring at STRC observation bed
3A. Clinical considerations*	Demographic	Age<br>Characteristics housing
	Somatic status**	Clinical status<br>Diagnosis-related<br>Plan for treatment
	Cognitive and mental status	Dementia<br>Delirium (or risk delirium)<br>Hallucinations<br>(Repellent) behavior<br>Other cognitive impairment(s)<br>Receptive to instruction(s) for rehabilitation<br>Awareness of illness<br>Momentary well-being
	Social***	Caregiver situation<br>Social system of patient<br>Involvement of case manager (dementia)
	Mobility and functional status	Mobility<br>Functional independence (ADL/iADL)<br>Functional decline (ΔADL/ΔiADL)<br>Barthel index<br>Premorbid activity limitation<br>High fall risks<br>Patient's capacity for training
	Multi-domain****	Case complexity<br>Judgement concerning probability of returning to own home<br>LTC indication (and on waiting list)<br>Patient has a rehabilitation need<br>Self-management ability<br>(Reduced) self-care
	Patient preferences***	Limited treatment policy<br>Wishes of the patient with respect to LTC admission<br>Follow-up care possible at same facility? (less transfers)
3B. Organizational considerations*	Micro-level: Care process at facility	Diagnostic procedures<br>Observational procedures<br>Execution of treatment (plan)<br>Capability and expertise of professionals<br>Multidisciplinary care<br>Coordination of care<br>Environmental factors

	Meso-level: Regional organisation of (acute) elderly care	Availability of intermediate care beds (and which models)<br>Possibilities of acute care in an outpatient or home setting<br>Availability of home care<br>Availability of support material/ instruments to use at home<br>Time of day when referral decision-making takes place
	Macro-level: Healthcare system factors	Patient's insurance<br>Pressure to discharge early<br>Reimbursement for provided care (not sufficient)<br>Costs for society / efficiency of care<br>Admission to care model possible directly after ED visit?
3C. Weighing considerations	Weighing clinical considerations	Based on comprehensive (geriatric) assessment<br>Final conclusion made by attending medical specialist<br>Joint agreement between healthcare professional involved
	Weighing clinical and organizational considerations	Organisational triage factors limit referral options<br>Next best option

** Renamed, in the Groot et al. [32]: clinical and organizational triage factors*
*** renamed, in de Groot et al. [32]: diagnoses, syndromes*
**** new category*
***** renamed, in de Groot et al. [32]: multi-domain tools and measures*
Abbreviations: ADL = activities of daily living; AGCH= Acute Geriatric Community Hospital; GR = geriatric rehabilitation; iADL = instrumental activities of daily living; LTC = long-term care; ED = emergency department; STRC = Short-Term Residential Stay
